# Differentiation between external and internal cuing: An fMRI study comparing tracing with drawing

**DOI:** 10.1016/j.neuroimage.2007.03.005

**Published:** 2007-06

**Authors:** E. Gowen, R.C. Miall

**Affiliations:** a Faculty of Life Sciences, Moffat Building, The University of Manchester, P.O. Box 88, Sackville Street, Manchester, M60 1QD, UK; b University of Birmingham, United Kingdom

## Abstract

Externally cued movement is thought to preferentially involve cerebellar and premotor circuits whereas internally generated movement recruits basal ganglia, pre-supplementary motor cortex (pre-SMA) and dorsolateral prefrontal cortex (DLPFC). Tracing and drawing are exemplar externally and internally guided actions and Parkinson's patients and cerebellar patients show deficits in tracking and drawing, respectively. In this study we aimed to examine this external/internal distinction in healthy subjects using functional imaging. Ten healthy subjects performed tracing and drawing of simple geometric shapes using pencil and paper while in a 3-T fMRI scanner. Results indicated that compared to tracing, drawing generated greater activation in the right cerebellar crus I, bilateral pre-SMA, right dorsal premotor cortex and right frontal eye field. Tracing did not recruit any additional activation compared to drawing except in striate and extrastriate visual areas. Therefore, drawing recruited areas more frequently associated with cognitively challenging tasks, attention and memory, but basal ganglia and cerebellar activity did not differentiate tracing from drawing in the hypothesised manner. As our paradigm was of a simple, repetitive and static design, these results suggest that the task familiarity and the temporal nature of visual feedback in tracking tasks, compared to tracing, may be important contributing factors towards the degree of cerebellar involvement. Future studies comparing dynamic with static external cues and visual feedback may clarify the role of the cerebellum and basal ganglia in the visual guidance of drawing actions.

## Introduction

Movement can be initiated in response to external stimuli and cues or through internally driven, self-initiated processes. Different areas of the brain are thought to be preferentially involved in each form of movement. Most notably, the basal ganglia have been proposed to be more important for internally cued and memory-guided movements (Crawford et al., 1989; Flowers, 1976; Jueptner and Weiller, 1998; Mushiake and Strick, 1995; Van Donkelaar et al., 1999, 2000), whereas the cerebellum is believed to play a more prominent role in externally cued movements (Jueptner et al., 1996; Jueptner and Weiller, 1998; Van Donkelaar et al., 1999, 2000). In addition, the basal ganglia and cerebellum project to specific thalamic regions that are also selectively active in internally driven or externally driven movements, respectively (MacMillan et al., 2004; Vaillancourt et al., 2003; Van Donkelaar et al., 1999, 2000). In turn, those regions within the thalamus that are active for internally generated movement demonstrate stronger connections with the dorsolateral prefrontal cortex (DLPFC) and the pre-supplementary motor area (pre-SMA) (Matelli and Luppino, 1996) both of which are also active during self-initiated movement and working memory (Curtis and D’Esposito, 2003; Deiber et al., 1999; Frith et al., 1991; Jahanshahi et al., 1995; Jenkins et al., 1994; Lau et al., 2004; Oliveri et al., 2001). In contrast, thalamic regions involved in external guidance project to the dorsal premotor cortex (Matelli and Luppino, 1996; Van Donkelaar et al., 1999) which appears concerned with visuomotor integration (Wise et al., 1997).

Having emphasised this dissociation, it should be mentioned that there remains a degree of overlap in the neural circuitry controlling externally and internally cued movements. The cerebellum can function during internally cued movements (Mushiake and Strick, 1993) and the basal ganglia and SMA in externally cued movements (Jueptner et al., 1997a,b; Vaillancourt et al., 2006, 2003). These findings can be explained by the presence of specific subcircuits within the cerebellar and basal ganglia systems that are specific for each movement type and which are superimposed on a background of overlapping functions (Van Donkelaar et al., 1999, 2000). For instance, Jueptner and Weiller (1998) concluded that both the cerebellum and basal ganglia are concerned with improvement in motor performance, whereas the basal ganglia are preferentially involved in the selection of appropriate movements and the cerebellum in monitoring the outcome of movements by comparing with sensory inputs. Furthermore, activation of DLPFC during self initiated movement may be largely due to attention to the selection of action rather due to the act of self-initiation per se (Jueptner et al., 1997a; Lau et al., 2004).

This distinction between internally and externally generated movements can also be observed following motor dysfunction. Performance during tracking tasks improves for cerebellar patients when vision of the target or hand is removed, highlighting the impaired use of external cues after cerebellar lesions (Van Donkelaar and Lee, 1994). Parkinson’s disease (PD) patients who have a dopaminergic deficit affecting the basal ganglia and fronto-striatal networks typically display deficits in internally generated movements that are improved with the use of external cues (Briand et al., 1999; Crawford et al., 1989; Flowers, 1976; Martin et al., 1994; Morris et al., 1996). PD patients also display graphical impairments, in particular a reduction in pen stroke size for both writing (micrographia) (Van Gemmert et al., 2001) and drawing (Longstaff et al., 2003; Vinter and Gras, 1998) that is alleviated with the use of external cues (Martin et al., 1994; Oliveira et al., 1997). The aim of our current work was to investigate whether a simple paradigm comparing tracing against drawing would elicit different areas of brain activation involved in externally or internally generated movements, respectively, and so provide a potentially useful behavioural paradigm in which to explore these issues.

Tracing depends on external cues from the existing template and from visual feedback to monitor the pen tip position in relation to the required line. Drawing on a blank page employs internal cues to a greater extent, guiding the hand to self-selected positions. The use of visual or eye position feedback may play a significant role only at certain key points in the drawing, for example when joining two lines to complete a square or triangle. Consequently, there may be greater eye–hand coupling during tracing that requires detailed comparison between the template and pen line, and therefore increased external guidance of the pen tip. In contrast drawing may impose greater demands on memory and planning processes. Therefore we would predict that tracing will result in greater activation of the cerebellum and premotor cortex, due to increased external guidance whereas drawing will activate areas involved in memory and internally guided movements such as the basal ganglia, pre-SMA and DLPFC. Furthermore, as tracing may encourage greater eye–hand coupling due to an increased demand for accuracy (Gowen and Miall, 2006) and the cerebellum is believed to be particularly involved in tasks that require eye–hand coordination (Miall, 1998; Miall et al., 2000, 2001) this would be a further reason to expect greater cerebellar activation in the tracing condition. However, it should be noted that the majority of research supporting a cerebellar contribution to eye–hand coordination has employed eye–hand tracking tasks that entail tracking a moving target. As tracing involves a stationary visual template, cerebellar involvement may differ between the two task types.

Previous behavioural work has demonstrated differences between tracing and drawing eye–hand coordination with and without visual cues, suggesting that the two forms of movement recruit different brain areas. Flanders et al. (2006) compared the kinematics of tracing a seen shape with subsequent drawing from memory of that shape. They observed highly similar patterns between the two tasks but compared to the tracing task, subjects spent more time in areas of tight curvature during drawing and proposed that this represented a strategy for learning and remembering the shape. Moreover, saccades are smaller and more frequent during tracing compared to drawing, indicating closer coupling between the eye and hand during tracing (Gowen and Miall, 2006). In addition, during combined eye–hand pointing, the timing between saccade and hand onset is closer for remembered as opposed to visual targets (Sailer et al., 2000; Van Donkelaar and Staub, 2000) suggesting that each movement type recruits a different neural substrate. These behavioural studies are supported by imaging data: Jueptner et al. (1996) also aimed to dissociate drawing and copying, and observed greater activation in the superior parietal lobe and cerebellar hemispheres, nuclei and vermis during eye–hand tracking of single lines when compared with drawing lines in any freely chosen direction. In the reverse contrast, greater activity was observed in the dorsal and ventral prefrontal cortex. Interestingly, basal ganglia activity did not differ between the drawing and copying tasks.

Our current work aims to extend these findings in four different ways. Firstly, participants drew well known but specified shapes so reducing the contribution of processes involved in decision making. Secondly, we used a more natural task with pencil and paper, in which participants could observe their hand, and thus one which is closer to conditions under which micrographia is observed. Thirdly, our task involves tracing along a line printed on the page whereas Jueptner and colleagues used a task more akin to dynamic eye–hand tracking, in which participants tracked the end of a retracting line by movement of a computer mouse. Fourthly, by contrasting conditions with eye motion, hand motion and both, we have attempted to dissociate which brain areas are more closely associated with eye–hand coordination than during tasks that involve the eye or hand alone. Consequently, we employed fMRI to examine which areas of the brain are differentially activated during tracing compared to drawing and whether these areas reflect the distinction between externally and internally guided movements. We hypothesised that the tracing vs. drawing paradigm would differentially activate cortico-cerebellar and cortico-basal ganglia pathways, respectively, providing a useful tool to further investigate diseases such as PD where graphical tasks are impaired.

## Materials and methods

### Participants

We tested 10 healthy volunteers (5 females) whose average age was 22.2 years (range, 18–31). All were right handed and had no previous or current history of neurological or ocular disease or general health problems. Each gave written informed consent to participate and the study was approved by a local ethical committee.

### Task stimuli

**Fig. 1:**
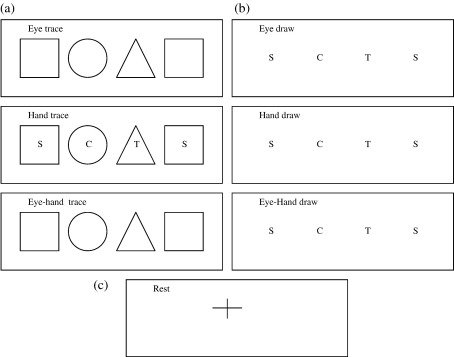
Examples of the 7 different conditions used in the experiment. The instruction is situated in the top left. Each condition represents one block and each was performed a total of 10 times, except the baseline condition that was performed 20 times. They were presented in the form of a booklet.

Subjects were required to trace around or draw three different shapes (square, circle, triangle) that were presented on a hand held booklet. The circumference of the square, circle and triangle were 24 cm, 18.85 cm and 18 cm, respectively. Each page on the booklet contained four shapes (hence one shape was repeated, in randomized order) and an instruction that indicated which of seven conditions should be performed (Fig. 1).

There were three tracing conditions (Fig. 1a):

Eye–hand tracing – tracing the outline of the printed shapes using both eyes and hand i.e. in the usual manner
Eye tracing – tracing the outline of shapes with eyes only
Hand tracing – tracing the outline of shapes with the hand only while the eyes were fixed on letter in centre of shape

For the drawing conditions, the shapes were omitted from the page and instead the first letter of each shape name (S, C, T) appeared on the sheet and prompted the subjects to draw the shapes around this letter (Fig. 1b):

Eye–hand drawing – drawing the outline of shapes in the usual manner i.e. using both eyes and hand
Eye drawing – moving the eyes to shift gaze around the path of the specified shapes
Hand drawing – drawing the specified shapes with the hand only (while the eyes were fixed on the instructing letter)

The final condition was baseline – fixating a central cross (Fig. 1c).

In both hand tracing and hand drawing conditions the instructing letters also served as the fixation point. The order of shapes in the booklet and the order of the tasks were counterbalanced across subjects. Therefore, there were six active conditions and one passive baseline condition. The three main factors in our design were task (tracing vs. drawing, method (eye vs. hand) and coordination (independent vs. coordinated).

### Experimental task

Each participant completed a safety screening form and was provided with the task instructions. A 20-min laboratory-based training phase was given prior to the experiment in order to familiarise the subjects with the different instructions and timings of the blocks and to verify that eye movements were performed in accordance with each condition. Subjects were then placed in the scanner with the booklet held in their left hand, comfortably resting on a pillow across their body, and with a pencil in their right hand. Subjects could view the booklet and their hand and pencil through a forward-facing, non-inverting mirror. Each block lasted 18 s and consisted of one of the seven conditions. Subjects were trained and instructed to perform the tracing or drawing task throughout each 18-s block and, if they finished the last shape prematurely, to return and redraw the first shape, reducing speed on subsequent blocks. In particular, they were instructed to deliberately and slowly move their eyes in the eye-only conditions. For the tracing task, they were instructed to trace the lines to their best ability, while in the drawing task they were instructed to reproduce accurate representations of the shapes that were of the same size as during the tracing task. Participants were not informed how to move their arm during the experiment but due to space restraints and instructions against large arm movements, movement was limited to the forearm, wrist and digits. Blocks were separated by 6-s periods where the subject turned the page ready for the next block. Altering the ambient light in the scanner with a data projector cued the subjects as to when they should turn the page: white = 18-s test condition, blue = 6-s page turning. Timing was controlled by Presentation (Neurobehavioral Systems) and was synchronized to the EPI volumes.

One run consisted of one repetition of each of the 7 conditions, except the baseline condition which was presented twice. Each subject performed 10 runs, separated into two 15-min scanning sessions of 5 runs each, giving a total of 80 blocks. The sessions were performed sequentially; a short 2-min break allowed a new booklet to be given to the subject.

### Functional imaging and analysis

For each subject 320 T2*-weighted fast echo-planar images were acquired in each 15-min session using a 3 T Philips scanner with an 8-channel parallel head coil and SENSE factor of 2.0 (TE = 35 ms, flip angle = 85°, TR = 3.0 s). Forty-nine interleaved slices provided whole brain coverage (acquisition matrix 96 × 96, FOV = 240 × 240 × 147 mm) with each voxel subtending 2.5 × 2.5 × 3 mm. Four dummy volumes preceded each of the two scanning sessions. High-resolution T1-weighted images were also acquired with 1 × 1 × 1 mm voxel size, 175 slices in sagittal orientation.

### fMRI processing and analysis

All fMRI signal processing and analysis was performed using the FMRIB software library (FSL version 5; FMRIB, Oxford). The initial four dummy volumes of each functional data collection run were discarded prior to analysis to ensure T1 saturation had been achieved. Prior to processing, slice timing was corrected and the volumes in each run were motion-corrected and realigned to the middle volume of the run using MCFLIRT. Maximum within scan head motion was less than 1.07 mm, and averaged 0.7 mm across the group. The BOLD signals were then high-pass filtered with a 48 s Gaussian-weighted filter, and spatially filtered with a 5 mm FWHM kernel.

Explanatory variables associated with each of the 6 active conditions were convolved with a gamma-derived haemodynamic response function (standard deviation of 3 s, mean lag of 6 s). The baseline fixation condition was not entered into the model so that all activation levels were calculated relative to this unmodeled condition. Epochs associated with page turning were entered into the GLM as a covariate of no interest. An additional variable was also included to model any blocks in which the subject failed to perform the correct task. Additionally, the motion correction parameters calculated by MCFLIRT were entered into the model as six covariates of no interest, without convolution by the HRF, and orthogonalized with respect to one another. Within each individual functional run, contrasts testing the factorial combination of the three main factors (task, method, and coordination) and their interactions were calculated.

At the second level of the analysis, contrasts were combined for each participant from the first-level analysis of the two functional imaging runs with a mixed effects treatment of the variance (FLAME stage 1 processing). The third level of the analysis combined the second level output across all participants (full FLAME processing). Voxels were initially thresholded at a *Z*-score value of 2.6 (equivalent to a *p* of .005, one-tailed), and then subjected to a cluster threshold with a significance level of *p* < .05.

Clusters of significant activity found from the group analysis were identified anatomically using comparisons between the 3dmrx (MRIcro) voxel labelled Brodmann atlas, an atlas for general neuroanatomical reference (Duvernoy and Bourgouin, 1999) and one for localisation within the cerebellum (Schmahmann et al., 2000). From the group average signal, a local maxima within these areas were compared across the 6 different active conditions using the Featquery tool (FMRIB, Oxford). Target voxels were identified as those of highest statistical significance observed in the specific group contrasts between conditions, or of individual conditions against baseline; Featquery then inverts the transformation used to register each individual’s brain into the MNI standard space in order to locate the voxel in the individual brain corresponding to the target.

## Results

### Behavioural analysis

**Fig. 2:**
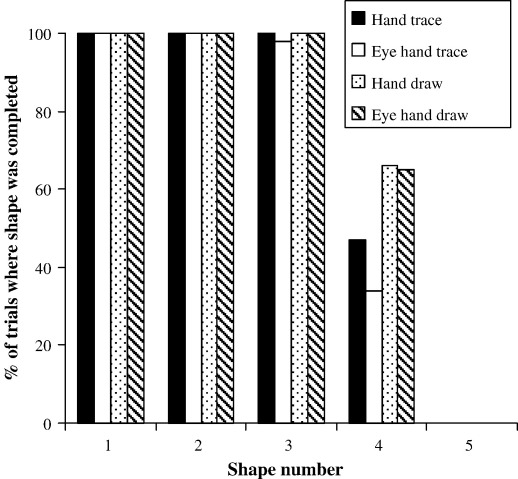
Graph depicting the % of trials where subjects completed 1–5 shapes across the four conditions of hand trace, eye–hand trace, hand draw and eye–hand draw. Shape 5 refers to recommencing at the beginning of the pad and re-drawing shape 1.

The average pathlength of the pencil motion for the reproduced shapes (square and triangle only) over the different conditions are shown in Table 1, column 1, measured directly off the paper. A between subject ANOVA with factors of drawing method (trace/draw) and degree of eye–hand coordination (eye–hand/hand) revealed a significant main effect of drawing method: shapes were smaller when drawn, as opposed to traced [*F*(1,76) = 29.24, *p* < 0.0001]. As an indication of the time taken to complete the drawing tasks, we calculated the % of completed shapes in each condition. Accurate timing would result in the initiation of less than 5 shapes (the 5th refers to re-drawing of the first shape), and the completion of more than 3. Subjects appeared well timed as they completed either three or four shapes (Fig. 2) and were found to re-trace or re-draw the first shape on less than 0.06% of all trials (Table 1, column 2). However, both hand drawing and eye–hand drawing were performed at a faster speed than the equivalent tracing conditions. These results are similar to our previous findings where subjects produced smaller and quicker drawings as opposed to tracings (Gowen and Miall, 2006).

### Functional activation

Our main comparisons of interest were between tracing and drawing tasks, and between tasks that involved independent use of the eye and hand (eye trace/draw, hand trace/draw) versus those tasks that employed coordinated use of the eye and hand (eye–hand trace/draw). In order to compare our data with previous work and to identify whether tracing and drawing provide a suitable paradigm in which to dissociate BG and cerebellar networks we have also included contrasts detailing both eye–hand tracing and drawing against baseline. Similarly, in order to verify that our paradigm was sensitive to the differences between the two drawing conditions we have also included a comparison of eye only vs. hand only tasks.

### Eye–hand tracing and drawing – baseline

The contrast between eye–hand tracing and baseline revealed significant activation in the cerebellar vermis VI and VIII, the right superior temporal pole and the right inferior frontal operculum (Table 2). Unexpectedly, the contrast between eye–hand drawing and baseline revealed similar activation in the cerebellar vermis VI and VIII, with the addition of the left Supplementary motor area and right superior parietal lobe/precuneus (Table 2). Significant BG activation was absent in both contrasts indicating that our tracing/drawing paradigm does not provide clearly differentiated BG activity.

#### Eye vs. hand

We next contrasted all eye-alone conditions (eye trace and eye draw, without hand motion) against hand-alone conditions (hand trace and hand draw, with eye fixation). For the eye tasks, activity was greater in left visual areas (BA 17, 18), left DLPFC (BA 46), left orbitofrontal cortex (BA 10), left ventral prefrontal cortex (BA 45), left ventral and dorsal premotor cortex (PMv, PMd) (BA 6), left inferior parietal (BA 39, 40), right FEF, right superior temporal sulcus (BA 22), right middle temporal gyrus (BA 21) and right putamen (Table 3). However, all areas of activation with the exception of BA 17, 18, PMd, PMv and FEF were caused by relatively greater deactivation in the hand conditions as they did not attain significance when the eye conditions were compared to baseline. In the reverse contrast (hand vs. eye), greater activation was observed in left primary motor cortex (BA 4), right posterior superior temporal sulcus, left somatosensory area (BA 3), left PMd (BA 6), right cerebellar lobules VII, VIII, right vermis VI, VII, VIII, right subcentral gyrus (BA 43), left fusiform gyrus (BA 37) and left inferior posterior parietal (Table 4).

These findings highlight the expected differences between the eye and hand tasks, where the eye tasks activate visual areas, and oculomotor circuits in FEF and premotor cortex (Grosbras et al., 2005; Heide et al., 2001; Konen et al., 2004; see Krauzlis, 2005 for a review) whereas the hand tasks favour activation of contralateral motor and premotor areas and ipsilateral cerebellar cortex (Grafton et al., 1992, 1996; Miall et al., 2001; Petit and Haxby, 1999; Simon et al., 2002).

#### Tracing vs. drawing

**Fig. 3:**
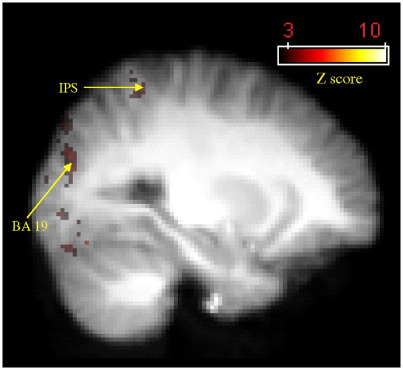
Activation map detailing areas of greater activity during hand tracing compared to hand drawing based on group data. Figure shows BA 19 and anterior intraparietal sulcus (IPS). Coordinates in MNI space are centred around the voxel of peak significance (*x* = − 26, *y* = − 48, *z* = 60). Colour bars indicate *Z*-score significance level, from the lowest score of 2.6 (red) to the highest score of > 10.0 (yellow).

**Fig. 4:**
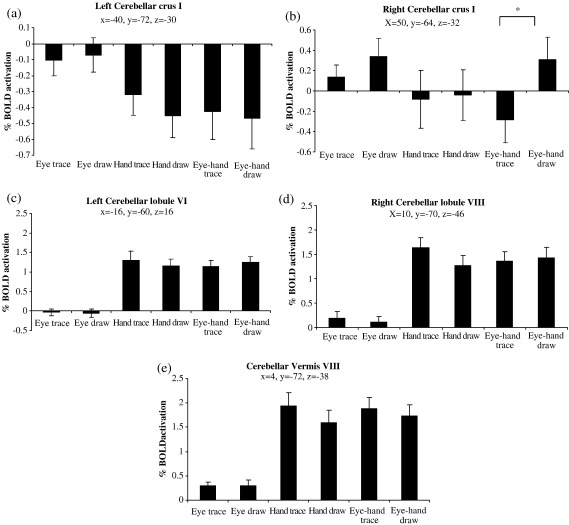
Percentage BOLD response of (a) left cerebellar crus I, (b) right cerebellar crus I, (c) left cerebellar lobule VI, (d) right cerebellar lobule VIII, and (e) cerebellar vermis VIII across the 6 conditions. Asterisk denotes significant difference between conditions (*P* < 0.001). Standard error bars are shown. Coordinates are in mm.

Firstly we compared all tracing conditions against all drawing conditions. We expected there to be greater activity in the cerebellum during tracing as opposed to drawing. However, only visual areas BA 17, 18 and 19 were significantly more active during the tracing conditions (Table 5). This was also found for both individual comparisons (eye trace versus eye draw, hand trace versus hand draw) but not for eye–hand tracing vs. eye–hand drawing, where no significant differences in activity were seen. Another area more significantly activated during hand tracing than hand drawing was the left anterior intraparietal sulcus (Fig. 3; Table 5). Fig. 4 displays mean group activation for 5 coordinates within the cerebellum across all 6 conditions. These loci were chosen as the voxels of locally maximum significance when contrasting all active conditions against baseline and sample the right crus I, lateral and vermal lobules VIII, and left crus I and lobule VI. It can be seen that for all areas except right crus I, cerebellar activity was stronger in all hand conditions and did not alter according to whether tracing or drawing was being performed. Right crus I appears to be preferentially activated during drawing conditions (see below).

**Fig. 5:**
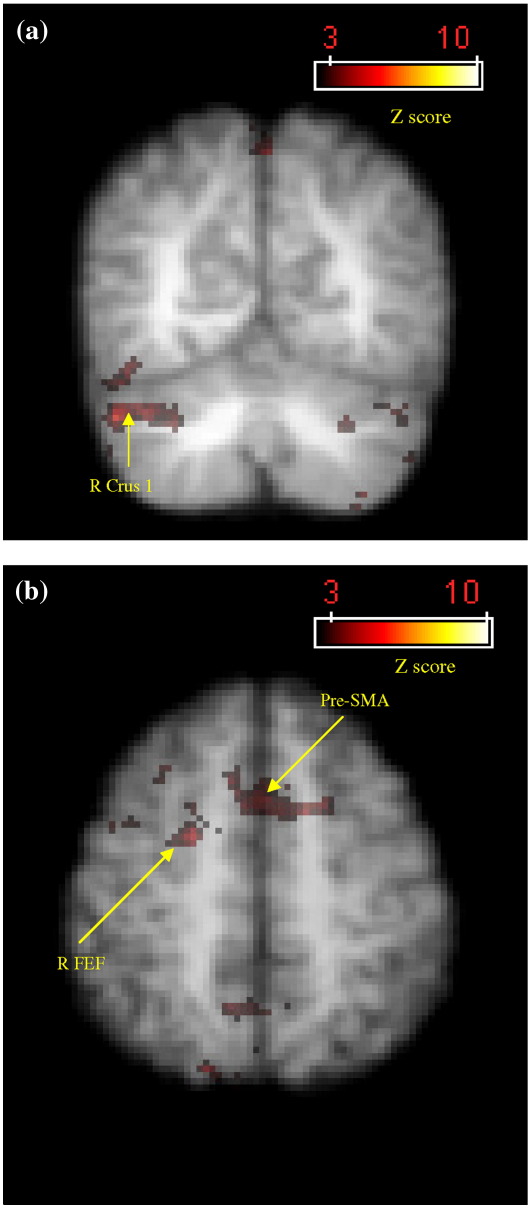
Activation map detailing areas of greater activity during eye–hand drawing compared with eye–hand tracing based on group data. (a) Right cerebellar crus I centred around the voxel of peak significance (*x* = 50, *y* = − 64, *z* = − 32). (b) Right FEF and pre-SMA centred around the voxel of peak significance for right FEF (*x* = 22, *y* = − 2, *z* = 54). Colour bars indicate *Z*-score significance level, from the lowest score of 2.6 (red) to the highest score of > 10.0 (yellow).

**Fig. 6:**
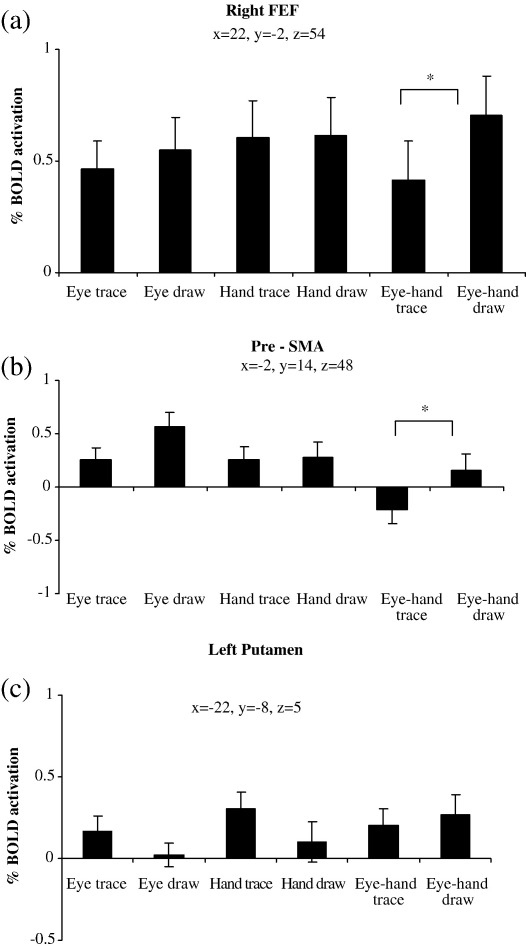
Percentage BOLD response of the (a) right FEF, (b) SMA and (c) left putamen across the 6 conditions. Asterisk denotes significant difference between conditions (*P* < 0.001). Standard error bars are shown. Coordinates are in mm.

In the reverse contrast where all drawing conditions were compared to all tracing conditions we expected greater activation in the basal ganglia, pre-SMA and DLPFC. We found no areas significantly more activated across all three drawing conditions. However, in the contrast between eye drawing vs. eye tracing, increased activation occurred in left anterior parietal and left PMd (BA 6) (Table 6), while in the contrast between eye–hand drawing and eye–hand tracing, greater activation was observed in right cerebellar crus I, right BA 19, PMd, FEF, superior parietal lobe/precuneus, and in bilateral pre-SMA and left precuneus (Figs. 5a, b; Table 6). The increase in activity for drawing compared to tracing conditions in the right cerebellar crus I, right FEF and left pre-SMA can be observed in Figs. 4b and 6a–b, respectively. It is interesting to note that activity in the right cerebellar crus 1 (Fig. 4b), FEF (Fig. 6a) and pre-SMA (Fig. 6b) actually decreased during the eye–hand tracing condition. No areas of the basal ganglia showed greater activation for the drawing as opposed to the tracing conditions. Moreover, activation was low in all areas of the BG, as demonstrated in Fig. 6c for the right putamen.

#### Eye–hand coordination

**Fig. 7:**
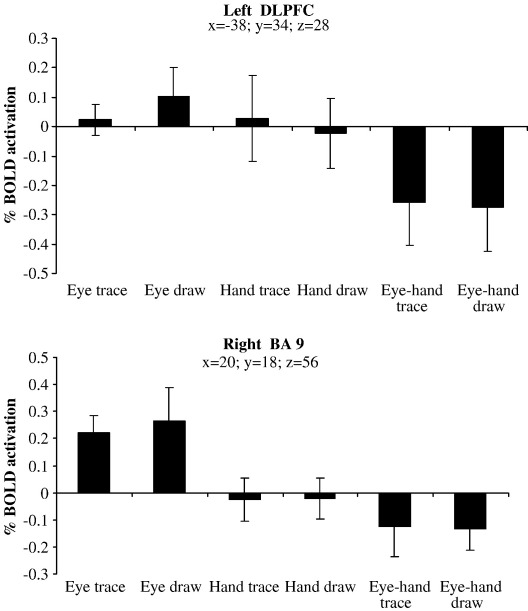
Percentage BOLD response of the (a) left DLPFC and (b) right BA 9 across the 6 conditions. Standard error bars are shown. Coordinates are in mm.

**Fig. 8:**
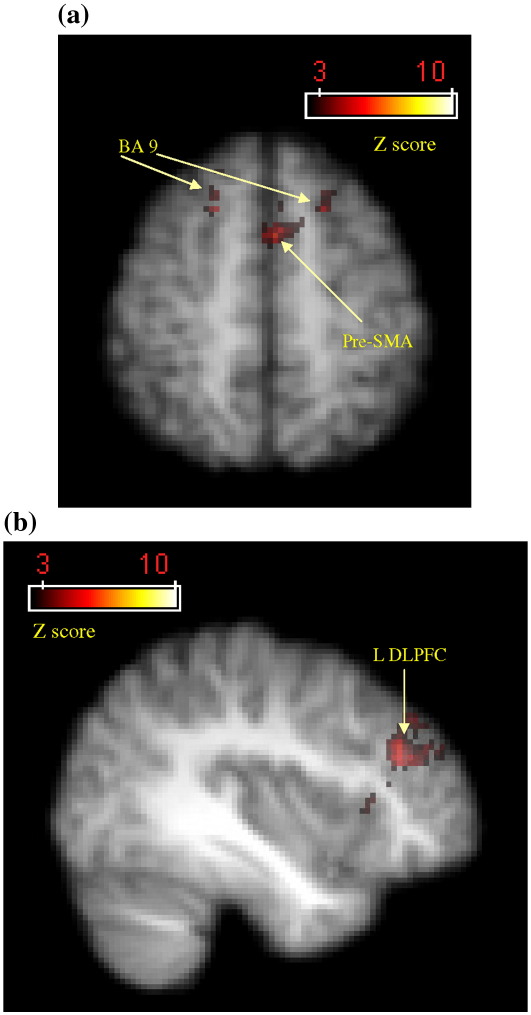
Activation map detailing areas of greater activity during independent drawing compared with coordinated eye–hand drawing based on group data. (a) Right and left BA 9 and pre-SMA activation centred around the voxel of peak significance for the pre-SMA (*x* = − 2, *y* = 8, *z* = 56). (b) Left DLPFC activity centred around the voxel of peak significance (*x* = − 38, *y* = 34, *z* = 28). Colour bars indicate *Z*-score significance level, from the lowest score of 2.6 (red) to the highest score of > 10.0 (yellow).

We expected more cerebellar activity in the conditions that involved using both the eye and hand to trace or draw (coordinated conditions) in contrast to those conditions where only the eye or hand were tracing or drawing (independent conditions). However, in the comparison between independent tracing (eye-trace, hand-trace) and combined tracing (eye–hand tracing), greater activation was observed in prefrontal areas (right and left BA 46 and right BA 47), as well as right superior temporal lobe (BA 22), right posterior IPS (BA 39), right BA 18 and the left cerebellum crus I (Table 7, top section). It can be observed from Figs. 4a and 7a that activation appeared greater in the left cerebellum and right DLPFC because these areas displayed deactivation in the combined eye–hand conditions, which was also the case for all the aforementioned areas. When compared against baseline, none of these areas were significantly activated. In the contrast examining coordinated tracing vs. independent tracing, differential activation was not apparent at any location. We observed greater activation in the left pre-SMA, right and left prefrontal areas (BA 9), right anterior cingulate sulcus and left DLPFC (BA 46) when comparing independent drawing to combined drawing (Figs. 7a–b and 8a–b). Although these areas exhibited deactivation in the combined eye–hand condition, they showed significant activation when the independent conditions were compared to baseline. No significant activations occurred in the reverse contrast of combined drawing vs. independent drawing. Cerebellar activity appeared to depend more on whether the hand was used in the task than whether the eye and hand were used in combination (Figs. 4a, c–e).

#### Summary

Overall, the tracing conditions recruited visual areas (BA 17, 19) to a greater extent than drawing conditions, but did not preferentially involve the cerebellum or PMd cortex. In contrast, drawing tasks recruited the right cerebellar crus I, right and left pre-SMA, right dorsal premotor cortex, right FEF, left precuneus and right superior parietal lobes/precuneus but did not preferentially activate the basal ganglia. Finally, in comparison to the independent conditions, eye–hand coordination during tracing or drawing did not preferentially activate any areas, including the cerebellum. Conversely, greater activation occurred in left pre-SMA, right and left BA 9, anterior cingulate sulcus and left DLPFC during independent drawing than combined drawing. Consequently, although our data do indicate that drawing activates a different set of neural areas to tracing, we found no evidence to suggest that the basal ganglia are more concerned with drawing or that the cerebellum is with tracing. Finally, the apparent deactivation in right FEF, pre-SMA and right cerebellar crus 1 during the eye–hand tracing task when compared to baseline suggests that combined eye–hand tracing leads to less involvement of these areas than any other task, including fixating a cross.

## Discussion

We examined whether the everyday eye and hand task of tracing or drawing shapes on paper would elicit different areas of brain activation that are involved in external compared with internal guidance of movement, respectively. In particular, we expected the cerebellum and premotor cortex to show more activation during tracing, and the basal ganglia, pre-SMA and DLPFC to be more active during drawing. When compared to baseline, our tasks showed activation similar to that reported in previous drawing type paradigms, namely in dorsal premotor, superior parietal and cerebellar regions (Lewis et al., 2003; Jueptner et al., 1996; van Mier et al., 1998). Although we found evidence that drawing and tracing do recruit different brain areas, cerebellar and basal ganglia activity was not modulated in the expected manner by either task. Indeed, the initial contrasts of eye–hand tracing/drawing – baseline demonstrate highly similar cerebellar activation in both tasks and no significant BG activity in the drawing task. Our main findings can be summarised as follows: (1) With the exception of visual areas, tracing did not recruit any additional brain areas compared to drawing and actually resulted in deactivation in the FEF, pre-SMA and right cerebellar crus I when compared to baseline. (2) Compared to tracing, drawing recruited greater activation of right cerebellar crus I, pre-SMA, PMd, right superior parietal/precuneus and left precuneus. (3) Coordinated eye–hand conditions did not activate any areas nor recruit additional areas more than in independent conditions, whereas the independent eye and hand conditions displayed greater activation in pre-SMA and prefrontal areas (BA 9, 46). We will address each of these findings in the following paragraphs.

### Comparison between tracing and drawing

#### Tracing tasks

The only areas to be more active in the tracing as opposed to drawing conditions were those striate and extrastriate areas concerned with visual processing (BA 17, 18, 19), and the anterior IPS which was more active during hand tracing than hand drawing. The increased visual activity coincides with the processing of the displayed visual templates, and the IPS is frequently activated in tasks that involve manual movements (Astafiev et al., 2003; Binkofski et al., 1998; Desouza et al., 2000; Macaluso et al., 2003; Simon et al., 2002). Interestingly, anterior IPS appears more concerned with fine motor movements rather than general reaching tasks (Binkofski et al., 1998; Simon et al., 2002) which could reflect the need for high spatial accuracy of the pen with respect to the template in hand tracing compared to drawing, where there is less requirement for positional accuracy. Furthermore, the anterior IPS is involved in attentive tracking of targets while fixating (Culham et al., 1998) and in visual selection processing (Wojciulik and Kanwisher, 1999), indicating that activity of this area in hand tracing may be due to the increased requirement for covert monitoring and visual selection of the pen tip seen in peripheral vision while maintaining central fixation, in order to compare its trajectory with the template.

The absence of increased cerebellar activity during tracing was unexpected as the cerebellum is thought to be involved in combining external sensory cues with action (Jueptner et al., 1996; Jueptner and Weiller, 1998; Van Donkelaar et al., 1999, 2000). A proposed function of the cerebellum is to overcome sensory feedback delays by producing a predictive estimate of the sensory outcome of movement which can then be compared to the external goal (Kawato et al., 2003; Miall et al., 1993; Miall and Wolpert, 1996; Wolpert et al., 1998). This enables planning errors to be rectified faster than if using visual feedback alone, so creating a smoother, more accurate movement. In a similar task to ours, Jueptner et al. (1996) did find greater activity in the left cerebellar hemispheres, nuclei and vermis when subjects tracked a line with a mouse cursor compared with drawing new lines. However, their task involved tracking a moving target, whereas our task consisted of tracing a stationary template. Several other paradigms have also explored cerebellar activity during visually guided tracking of moving targets in healthy participants (Vaillancourt et al., 2003) and in cases of cerebellar damage or deactivation (Miall et al., 1987; Van Donkelaar and Lee, 1994). In such a tracking task, continual comparison between the moving target and the cursor places more timing and predictive demands on the ocular and manual control systems and therefore perhaps, greater cerebellar involvement. Although we did not directly compare tracking with tracing we speculate that as our task used shapes that were familiar, static and frequently repeated this could have reduced the subjects’ dependence on the external template and removed the temporal constraint of tracing at a specific rate. Activity within the cerebellum has been shown to decrease with increasing task familiarity (van Mier et al., 1998). In regard to the static nature of our tracing templates, different modes of control are apparent for static as opposed to dynamic tracking, as cerebellar patients show deficits for the latter but not the former (Van Donkelaar and Lee, 1994). In addition saccadic errors produced by cerebellar patients appear less apparent during pointing then tracking tasks (Sailer et al., 2005). These authors suggested that compared to tracking, pointing tasks involve less integration of proprioceptive hand information with visual input. Furthermore, cerebellar activity is reduced during conditions where visual feedback frequency is low, suggesting that the temporal frequency of visual feedback affects the manner is which external stimuli are processed (Vaillancourt et al., 2006). If more complex and unfamiliar shapes had been used, with greater emphasis placed on accuracy we may have seen an increase in cerebellar activity. The observation that, compared to baseline, tracing actually resulted in a decrease in activity in the FEF, pre-SMA and right cerebellar crus I indicates that demand on these areas was minimal.

#### Drawing tasks

In our comparison between drawing and tracing, greater activation was observed in the bilateral pre-SMA and PMd, right FEF and precuneus/superior parietal cortex, left precuneus and right cerebellar crus I. Whereas SMA-proper is more involved in movement execution, pre-SMA appears to be consistently activated during more cognitively demanding tasks such as those that involve movement preparation, self-generation and planning and memorising sequences of movements (Curtis and D’Esposito, 2003; Deiber et al., 1999; Heide et al., 2001; Grosbras et al., 2001; Jueptner et al., 1996; Lau et al., 2004; Lee et al., 1999; Ogawa et al., 2006; Picard and Strick, 1996, 2001) indicating that this area does distinguish between the internal vs. external nature of drawing and tracing.

The FEFs appear to be involved in preparing eye movements (Connolly et al., 2002), in covert attention shifting (Grosbras and Paus, 2002; Moore and Fallah, 2001, 2004; Smith et al., 2005; Thompson et al., 2005) and in producing memory-guided saccades (Gaymard et al., 1999; Muggleton et al., 2003; Ozyurt et al., 2006). Therefore, increased FEF activity observed during drawing may have been due to greater planning and attention demands involved in producing saccades to an undefined, internally chosen goal. This is reflected in behavioural data where saccades are larger and less frequent during drawing than tracing (Gowen and Miall, 2006).

The PMd was also more active during drawing than tracing. PMd is composed of two main sections: a caudal section (PMdc or PMd proper) and a rostral section (PMdr or pre-PMd) (Boussaoud, 2001; Picard and Strick, 2001). PMdc is concerned with hand movement preparation and execution (self-paced finger movement, object manipulation), whereas PMdr plays a greater role in more cognitively demanding tasks (imagined finger movement, spatial attention shifting, memory, mental calculations) and eye movements (Battaglia-Mayer et al., 2001; Boussaoud, 2001; Fujii et al., 2000; Hanakawa et al., 2003; Picard and Strick, 2001). Interestingly, the PMd activity we observed in the eye conditions (Table 3) was more rostral compared to that for the hand conditions (Table 4). In addition, the coordinates for the locus of maximum activation in both eye drawing and eye–hand drawing (Table 6) also appear more rostral to the coordinates for all hand tasks, suggesting that drawing tasks were preferentially activating PMdr. No difference in PMd activity was observed between tracking and drawing lines in the study by Jueptner et al. (1996). Their task consisted of drawing single line segments in a self-chosen manner without recalling the line to be drawn, suggesting that the PMdr activity seen in our task may instead reflect recalling and visualising the spatial configuration of the shape to be drawn.

One puzzling finding is that the right cerebellar crus I was actually more strongly activated during drawing than tracing. Recent work has highlighted a cognitive role for the lateral cerebellum in functions such as planning, set shifting, working memory, abstract reasoning and linguistic skills (Schmahmann, 2004). In particular, involvement of the cerebellar crus I has been reported in non-motor attention tasks that involve attending to a stimulus (Allen et al., 1997) or task shifting (Le et al., 1998) and in visuospatial working memory (Nitschke et al., 2004). Of special interest are the findings of Nitschke and colleagues where cerebellar crus I as well as lobules VIIb and VIII showed preferential activity for memorised saccades compared to visually guided saccades.

Interestingly, activity differentiating eye–hand drawing from eye–hand tracing appears to be mainly in the right hemisphere of the FEF, PMdr, cerebellar crus I and parietal lobe. The FEF, PMdr and parietal lobe are involved in spatial attention shifting, and one speculation is that more attention (and eye movements) needs to be directed leftward when drawing a series of shapes, from left to right across the page, in order to correctly position the new shape and prevent overlap; the fixed location of the templates in the tracing condition avoids this requirement. How this may be related to the right cerebellar crus I activity is currently unclear to us; it is ipsilateral to the moving hand, and so might reflect activity related to eye–hand interactions that are more evident in drawing than tracing. However, behavioural data suggest the opposite pattern, with greater interaction in tracing (Gowen and Miall, 2006), and more work will be needed to resolve this issue.

In contrast to the results of Jueptner et al. (1996), we failed to observe greater activity in prefrontal cortex (BA 9, 46, 45, 47) during the drawing task. The activation of prefrontal cortex in their study may be attributed to the requirement to choose at will the line direction to be drawn, whereas in our study subjects were cued to draw well known and simple, predefined shapes. As subjects tend to draw these shapes in highly stereotyped fashion, they would be unlikely to be making a free choice of direction or line segment. This is supported by evidence displaying that DLPFC activity is associated with decision processes and action selection (such as what action to perform and when it should be performed) as opposed to the generation of internal actions per se (Jueptner and Weiller, 1998; Lau et al., 2004; Playford et al., 1992). Finally, as we contrasted all our conditions against a fixation task (baseline) that is known to generate activation in prefrontal cortex (BA 9, 46, 45, 47) (Anderson et al., 1994; Jueptner et al., 1996), any prefrontal activation during the active drawing condition may have been less than during fixation and so removed in the contrast.

One aspect where our study complements the findings of Jueptner and colleagues is the observation that basal ganglia activity did not differentiate between tracing and drawing, although their task appeared to produce much stronger activation than our own. They further observed that right putamen activation was greater during fixation than when simply pursuing a contracting line and as our conditions were all initially contrasted against fixation this may explain the relatively low signal in the putamen at least. However, this choice of baseline would not obscure any differential activation between drawing and tracing. Involvement of the basal ganglia in internally driven tasks has not been consistently found (Mushiake and Strick, 1993; Jueptner et al., 1997a,b; Vaillancourt et al., 2003) suggesting that the dissociation between the cerebellum and basal ganglia in regard to externally and internally guided movements is not complete. Indeed, the basal ganglia are comprised of different cortico-basal ganglia circuits that respond differently to various demands such as task complexity and frequency (Lehericy et al., 2006) and therefore, the different findings across studies may reflect the functions of these specific sub circuits. Activity in the caudate and anterior putamen appears to be greater for complex movements (Lehericy et al., 2006) and in selection of appropriate movements (Jueptner and Weiller, 1998) but lower during the performance of prelearned tasks (Jueptner et al., 1997a,b; although see Jenkins et al., 1994 for a contrasting result). Perhaps if our task had involved less familiar shapes that were repeated infrequently during the scan, differential activity might have been seen between tracing and drawing, because selection of appropriate movements would have been more critical while drawing an unfamiliar shape. Furthermore, it has also been shown that the BG play a stronger role during tasks that require feedforward control, such as in open loop situations where visual feedback of the effector is not available or when the task is easier (Ogawa et al., 2006; Seidler et al., 2004). Feedforward control may have been used equally for tracing and drawing due to the familiar and repetitive nature of the shapes. Indeed, eye–hand coupling during tracing decreases with more familiar shapes (Gowen and Miall, 2006) suggesting greater emphasis on feedforward control. It is perhaps surprising that basal ganglia activity did not parallel that of the pre-SMA as both structures are interconnected (Lehericy et al., 2004). It could be that although similar mechanisms were being used to execute both tracing and drawing, pre-SMA activity was specifically required for the self-initiated drawing task. Alternatively, pre-SMA activity appears to increase with the speed of movement (Deiber et al., 1999) and drawing was performed at a quicker pace than tracing, so the pre-SMA activity may be related to performance times. Indeed, the fact that average drawing speed was faster than that of tracing represents a potential confounding factor of our study, since without direct measures of the pencil motion, we can only estimate average performance. There are numerous reports documenting increased activation of sensorimotor and cerebellar areas as rate or speed of movement increases (Jancke et al., 1998a,b, 1999; Lewis et al., 2003; Turner et al., 2003) rendering it difficult to conclude whether the activation present during drawing compared to tracing is a function of task or speed. We note however that average tracing size was 18% larger than in drawing, so the increased rate of completed drawings of a smaller average size will have reduced the overall difference in velocity between the two conditions. Moreover, this does not affect our main finding that drawing and tracing cause indistinguishable activation of the basal ganglia.

#### Comparison between coordinated and independent eye–hand conditions

We attempted to isolate those brain areas that may play a more significant role in combined eye–hand tasks than those that use the eyes or hand independently. The cerebellum is one area known to contribute to coordinated movements between the eye and hand (Miall et al., 2000, 2001; Van Donkelaar and Lee, 1994) and as tracing requires increased control and accuracy we anticipated that cerebellar activation would be greatest in the eye–hand tracing condition. However, although cerebellar activity was minimal during eye tracing and drawing, confirming previous findings (Jueptner et al., 1996), no area of the cerebellum was preferentially involved in either eye–hand tracing or eye–hand drawing when compared to the independent eye or hand tasks of tracing or drawing. This suggests that cerebellar areas involved in eye–hand coordination tasks are also used for hand tasks performed independently of the eyes. In support of this, Miall et al. (2001) observed significant cerebellar activity in crus I, lobules VII and VIII during both coordinated and independent eye–hand tracking. Such activity may be in response to the increased amount of attention and control required in independent conditions in order to overcome natural tendency for hand to follow eyes (Allen et al., 1997).

A larger number of areas were active during the independent drawing compared to the combined drawing conditions, suggesting that the unnatural nature of the task demanded higher processing. This is highlighted by increased activity of dorsal prefrontal areas and pre-SMA which, as detailed earlier, are often involved in more cognitively challenging tasks. The increase in pre-SMA (Fig. 6b) and left DLPFC (Fig. 7a) activity appeared to be related more specifically to eye drawing. This complements the role of the DLPFC in the control of memory-guided and predictive saccades (Pierrot-Deseilligny et al., 2003, 2005) and of the pre-SMA in the production of new saccade sequences (Grosbras et al., 2001), as one would expect that drawing a shape with the eyes only would be an unfamiliar challenge in comparison to the normal task of combined eye–hand drawing, and even tracing a line with the eyes. Activation of rostral areas of the cingulate cortex has been linked to self-initiated movement, new learning, error detection and action and outcome monitoring (Deiber et al., 1999; Jueptner et al., 1997a,b; Lau et al., 2004; Picard and Strick, 1996; see Rushworth et al., 2004 for a review) any of which could have been important during independent hand drawing. It is interesting to note that none of the areas more active in the contrast of independent versus coordinated tracing (Table 6) were also more active in the independent tracing condition relative to baseline. This highlights that even in the more demanding independent conditions, activation during tracing is still of equivalent magnitude to that seen when fixating a cross.

## Conclusions

Our aim was to identify whether the everyday tasks of tracing and drawing using a pencil and paper could differentiate between neural circuits involved in external and internal movement generation, respectively. It is clear that compared with tracing, drawing does recruit additional brain areas that play a stronger role in more cognitively challenging functions such as the control of self-initiated movements, spatial memory and spatial attention. However, activation in the areas traditionally most associated with the external/internal distinction such as the cerebellum and basal ganglia were not modulated in the expected manner, suggesting that the distinction between internal and external guidance is minimised when moving in these simple, repetitive and static paradigms and that subjects used similar strategies for both tracing and drawing. Thus consideration should be given to task familiarity, difficulty level and whether the visual cues are stationary or moving. In previous contrasts between internal and external guidance, the external cues usually impose implicit or explicit temporal constraints. Tracing a dynamic, moving, unfamiliar template may increase reliance on visual feedback and thus lead to differential cerebellar activation. Similarly, tasks requiring drawing of more complex unfamiliar shapes may invoke greater basal ganglia involvement to select the appropriate movements. However, our study suggests that in the everyday task of tracing or drawing simple, highly familiar shapes, there are insufficient differences to fully tax either internal or external guidance systems. Further work will be needed to understand the micrographic symptoms of Parkinson’s disease.

## Figures and Tables

**Table 1:** Mean pencil pathlengths and % of trials where participants finished all four shapes and returned to the first shape for 4 different conditions

	Mean pathlength ± SD, cm (target = 21 cm)	% of restart trials
Hand trace	20.35 ± 2.73	0.00
Eye–hand trace	21.025 ± 2.67	0.02
Hand draw	17.525 ± 2.12	0.06
Eye–hand draw	17.6 ± 2.54	0.04

Pathlengths were calculated for the square and triangle only; the mean length of the printed templates was 21 cm.

**Table 2:** Areas activated during eye–hand tracing – baseline (top section) and during eye–hand drawing – baseline (bottom section)

Area	Cluster volume (mm^3^)	Cluster *P*	*Z*	Laterality	Coordinates (mm)		
*x*	*y*	*z*
*Eye–hand trace – baseline*							
Cerebellar vermis VI	562.30	< 0.0001	9.18	R	4	− 62	− 16
Cerebellar vermis VIII			9.16	R	2	− 74	− 36
Dorsal premotor cortex (BA 6)			7.49	L	− 26	− 18	58
Primary motor cortex (BA 4)			7.34	L	− 34	− 28	62
Superior temporal pole (BA 38/22)	9.7	0.03	5.03	R	52	6	− 2
Inferior frontal operculum (BA 48)			4.62	R	48	− 2	2

*Eye–hand draw – baseline*							
Cerebellar vermis VI	667.7	< 0.0001	9.77	R	4	− 62	− 16
Cerebellar vermis VIII			8.82	R	4	− 72	− 38
Dorsal premotor cortex (BA 6)			8.9	L	− 28	− 26	70
Precuneus /Superior parietal lobe			8.73	R	12	− 64	− 50
Supplementary motor area			8.51	L	− 4	− 22	48
Somatosensory area (BA 3)			7.85	L	− 36	− 30	50
Primary motor cortex (BA 4)			7.76	L	− 34	− 28	62

**Table 3:** Areas more activated during eye conditions (eye trace/draw) than during hand conditions (hand trace/draw)

Area	Cluster volume (mm^3^)	Cluster *P*	*Z*	Laterality	Coordinates (mm)		
*x*	*y*	*z*
*All eye conditions – all hand conditions*							
Primary visual cortex (BA 17)	251.7	< 0.0001	10.6	L	− 10	− 80	10
Extrastriate visual cortex (BA 18)			10.3	L	− 8	− 92	12
Orbitofrontal cortex (BA 10)^a^	150.17	< 0.0001	7.03	L	− 44	52	10
Ventral prefrontal cortex (BA 45)^a^			6.5	L	− 52	36	18
Ventral premotor cortex (BA 6)			6.46	L	− 58	14	10
Dorsal lateral prefrontal cortex (BA 46)^a^			5.97	L	− 44	40	26
Inferior parietal (BA 40)^a^	45.56	< 0.0001	8.48	L	− 62	− 50	44
Inferior parietal (BA 39)^a^			5.96	L	− 44	− 68	30
Dorsal premotor cortex (BA 6)	29.47	< 0.0001	6.46	R	50	8	40
FEF (BA 8)			5.87	R	46	− 12	46
Superior temporal sulcus (BA 22)^a^	20.53	0.001	7.11	R	60	− 30	8
Middle temporal gyrus (BA 21)^a^			5.98	R	56	− 22	− 2
Putamen^a^			4.22	R	26	14	0

a Areas where apparent activation is due to relative deactivation in hand conditions.

**Table 4:** Areas more activated during hand conditions (hand trace/draw) than during eye conditions (eye trace/draw)

Area	Cluster volume (mm^3^)	Cluster *P*	*Z*	Laterality	Coordinates (mm)		
*x*	*y*	*z*
*All hand conditions – all eye conditions*							
Primary motor cortex (BA 4)	198.66	< 0.0001	19.8	L	− 38	− 30	60
Posterior superior temporal sulcus			4.79	R	50	− 40	16
Dorsal premotor cortex			8.64	L	− 26	− 18	68
Somatosensory area (BA 3)			8.55	L	− 44	− 28	58
Cerebellar lobule VII	196.05	< 0.0001	10.4	R	8	− 74	− 46
Cerebellar vermis VIII			10.3	R	4	− 70	− 34
Cerebellar lobule VIII			9.55	R	10	− 72	− 50
Cerebellar vermis VII			9.44	R	4	− 68	− 26
cerebellar Vermis VI			9.34	R	4	− 66	− 22
Subcentral gyrus (BA 43)	39.38	< 0.0001	6	R	68	− 14	30
Fusiform gyrus (BA 37)	22.97	0.0005	6.85	L	− 46	− 70	6
Inferior posterior parietal (BA 39)			4.22	L	− 42	− 82	20

**Table 5:** Areas more activated during tracing compared with drawing

Area	Cluster volume (mm^3^)	*P*	*Z*	Laterality	Coordinates (mm)		
*x*	*y*	*z*
*All tracing vs. drawing conditions*							
Primary visual cortex (BA 17)	51.34	< 0.0001	5.19	R	14	− 84	0
Superior occipital lobe (BA 18)			4.32	L	− 8	− 76	− 8
Inferior occipital lobe (BA 19)			4.53	R	24	− 96	0
		4.5	L	− 34	− 80	8

*Hand trace vs. hand draw*							
Inferior occipital lobe (BA 19)	53.1	< 0.0001	5.34	L	− 40	− 82	20
Posterior parietal (anterior intraparietal sulcus)	10.01	0.03	3.81	L	− 26	− 48	60

Top section of table compares all tracing conditions (eye tracing, hand tracing, eye–hand tracing) against all drawing conditions (eye drawing, hand drawing, eye–hand drawing) and bottom section compares hand trace against hand draw.

**Table 6:** Areas more active during eye drawing than eye tracing

Area	Cluster volume (mm^3^)	*P*	*Z*	Laterality	Coordinates (mm)		
*x*	*y*	*z*
*Eye drawing vs. eye tracing*							
Anterior parietal area (BA 3)	11.45	0.01	4.05	L	− 60	− 10	40
Dorsal premotor cortex (BA 6)			3.96	L	− 54	− 2	42

*Eye–hand drawing vs. eye–hand tracing*							
Cerebellum crus I	31.11	< 0.0001	5.58	R	50	− 64	− 32
Inferior occipital lobe (BA 19)			5.33	R	42	− 72	− 14
Pre-supplementary motor area (BA 6)	30.38	< 0.0001	5.26	L	− 2	14	48
		4.4	R	8	12	44
Dorsal premotor cortex (BA 6)			4.45	R	22	− 2	54
FEF (BA 8)			4.45	R	22	− 2	54
Precuneus	10.54	0.01	4.36	L	− 6	− 70	58
Precuneus /Superior parietal lobe			4.25	R	18	− 76	50
Superior occipital lobe (BA 18)			3.78	R	22	− 72	32

Top section of table shows areas more active during eye drawing than eye tracing and bottom section those areas more active during eye–hand drawing than eye–hand tracing.

**Table 7:** Areas more active during independent eye conditions than during coordinated eye–hand conditions

Area	Cluster volume (mm^3^)	*P*	*Z*	Laterality	Coordinates (mm)		
*x*	*y*	*z*
*Independent tracing–coordinated tracing*							
Dorsal prefrontal cortex (BA 46)^a^	410.66	< 0.0001	7.35	R	42	36	34
Ventral prefrontal cortex (BA 47)^a^			7.13	R	30	24	− 20
Dorsal prefrontal cortex (BA 46)^a^			6.32	L	− 32	38	38
Superior temporal lobe (BA 22)^a^			6.08	R	60	− 50	22
Inferior parietal lobe (posterior intraparietal sulcus) (BA 39)^a^			5.89	R	48	− 76	32
Extrastriate visual cortex BA 18^a^			5.75	L	− 6	− 96	28
Cerebellar crus I^a^	21.58	0.0008	4.63	L	− 40	− 72	− 30

*Independent drawing–coordinated drawing*							
Pre-supplementary motor area (BA 6)	17.46	0.004	5.69	L	− 2	8	56
Prefrontal cortex (BA 9)			4.7	L	− 20	18	56
		4.24	R	24	28	56
Cingulate sulcus (anterior cingulate motor areas)			4.09	R	14	30	34
Dorsal lateral prefrontal cortex (BA 46)	11.83	0.03	5.64	L	− 38	34	28

Top section compares independent tracing against coordinated tracing and bottom section compares independent drawing against coordinated drawing.

a Areas where apparent activation is due to relative deactivation in coordinated conditions.
